# Standard-of-care vs. machine learning-recommended discharge destinations for geriatric surgical inpatients: algorithm development and internal validation

**DOI:** 10.1007/s41999-026-01566-z

**Published:** 2026-07-31

**Authors:** Thomas Derya Kocar, Utz Lovis Rieger, Filippo Maria Verri, Pauline Maier, Christoph Leinert, Simone Brefka, Christian Bolenz, Nuh Rahbari, Florian Gebhard, Dhayana Dallmeier, Michael Denkinger, Hans Kestler

**Affiliations:** 1 Institute for Geriatric Research Ulm, Ulm University Medical Center, Zollernring 26, 89073 Ulm, Germany; 2 Agaplesion Bethesda Clinic Ulm, Ulm, Germany; 3 Institute of Medical Systems Biology, Ulm University, Ulm, Germany; 4 Department of Urology and Pediatric Urology, Ulm University Medical Center, Ulm, Germany; 5 Department of General and Visceral Surgery, Ulm University Medical Center, Ulm, Germany; 6 Department of Orthopedic Trauma, Hand, Plastic and Reconstruction Surgery, Ulm University Medical Center, Ulm, Germany; 7 Department of Epidemiology, Boston University School of Public Health, Boston, MA USA

**Keywords:** Discharge planning, Machine learning, Artificial intelligence, Geriatrics, Geriatric co-management, Post-acute care

## Abstract

**Purpose:**

Older adults undergoing surgery are at high risk for adverse outcomes due to age-related vulnerabilities. Comprehensive geriatric assessment (CGA) can improve discharge planning but is often limited by resource constraints. We evaluated whether machine learning (ML) can support individualized discharge destination recommendations for geriatric surgical inpatients.

**Methods:**

We developed and internally validated an AdaBoost classifier using electronic health record and CGA data from 169 patients aged ≥ 70 years undergoing surgery at a single center. The model predicted discharge destinations (home, acute geriatric care unit, rehabilitation facility, nursing home) and was evaluated using accuracy, receiver operating characteristic (ROC) curves, and calibration within a fivefold cross-validation framework. Model performance was also compared with previously reported standard-of-care discharge decisions.

**Results:**

The study population consisted of 169 older adults, with mean age of 80.5 years (SD ± 6.3). The ML model achieved a micro-averaged ROC area under the curve of 0.94 [95% CI 0.92–0.96] and an accuracy of 82% [95% CI 0.76–0.86], representing a significant improvement over standard-of-care decisions (138 vs 124 correct, *p* = 0.041). Mimicking a human-in-the-loop strategy, we excluded the 15% of predictions with the highest uncertainty, which increased accuracy for the remaining predictions to 85% (*p* = 0.006). Model inspection identified Barthel Index, Clinical Frailty Scale, age, and number of medications as key predictors.

**Conclusion:**

Our findings provide a proof-of-concept that an ML-based approach can effectively support individualized discharge planning for geriatric surgical patients. With 82% congruence with expert recommendations, the model significantly outperformed the standard of care. Its real-world clinical impact is currently being evaluated in an ongoing interventional trial.

**Trial registration:**

German clinical trials registry (DRKS00030684), registered on 21st November 2022.

**Supplementary Information:**

The online version contains supplementary material available at https://doi.org/10.1007/s41999-026-01566-z.

## Key summary points

**Aim:**

To investigate the potential of machine learning to support individualized discharge destination recommendations for older adults undergoing surgery.

**Findings:**

A machine learning algorithm predicted discharge destinations with 82% accuracy, increasing to 85% in a human-in-the-loop approach. These results demonstrate a significant improvement over standard-of-care decisions (*p* = 0.041 and *p* = 0.006, respectively).

**Message:**

Machine learning-based decision support offers a scalable and reliable tool to enhance discharge planning for geriatric surgical patients, helping align recommendations with expert clinical judgment and potentially improving clinical outcomes.

**Supplementary Information:**

The online version contains supplementary material available at https://doi.org/10.1007/s41999-026-01566-z.

## Introduction

Older adults represent a growing proportion of the surgical population, posing substantial challenges for healthcare systems. Age-related conditions such as multimorbidity, frailty, and cognitive decline are common in this group and substantially increase the risk of postoperative complications, prolonged hospitalization, institutionalization, and mortality [1]. Geriatric co-management, particularly through a comprehensive geriatric assessment (CGA), addresses these complex needs by providing structured, multidisciplinary care. Robust evidence shows that CGA reduces mortality and inappropriate discharge to higher levels of care, thereby supporting functional independence in older adults [2]. Orthogeriatric co-management after hip fracture is associated with reduced short- and medium-term mortality, albeit sometimes at the cost of longer hospital stays [3]. Similar benefits have been demonstrated in cardiology and trauma populations, including improved discharge disposition and access to rehabilitation services [4, 5]. Despite these benefits, widespread implementation of geriatric co-management remains limited, primarily due to a shortage of geriatricians and constrained resources [6]. Digital decision support and artificial intelligence (AI) therefore offer a promising, scalable approach to extending geriatric expertise beyond traditional service models.

Discharge planning is a cornerstone of high-quality surgical care in older adults, with major implications for functional recovery, continuity of care, and healthcare utilization. Poorly coordinated transitions are associated with functional decline, increased readmissions, and loss of independence, disproportionately affecting frail older adults [7, 8]. Structured discharge planning and referral to geriatric rehabilitation or transitional care services improve functional outcomes, reduce nursing home admission, and lower readmission rates [9–12].

The SURGE-Ahead project was established to address these gaps in geriatric care by integrating geriatric co-management with digital decision support across the surgical care pathway [13]. Within this framework, our working group demonstrated that 27% of standard-of-care discharge decisions for geriatric surgical inpatients were incongruent with geriatric expert recommendations derived from CGAs [14]. Crucially, these mismatches were associated with greater functional decline and higher 3 month readmission rates, thereby explicitly identifying suboptimal discharge decision-making as a modifiable contributor to adverse outcomes. Our findings highlight the need for scalable, data-driven tools to support individualized discharge planning within geriatric surgical care.

Machine learning (ML) offers a promising approach to meet this need. Predictive models based on electronic health record (EHR) data have shown promising results predicting discharge destination and timing [15–17], with downstream benefits including optimized post-acute care referrals and reduced readmissions [18]. However, these studies neither incorporate a CGA nor relied on predefined clinical decision rules. Compared with sensor-based or deep learning approaches that were previously explored within the SURGE-Ahead project [19], EHR- and CGA-based models are more feasible, interpretable, and readily integrated into routine clinical workflows. Well-calibrated, explainable, and uncertainty-aware algorithms can provide probabilistic discharge recommendations and trigger human interventions, such as geriatric consultation, when prediction uncertainty is high.

Building on the SURGE-Ahead cohort and its prior findings, the present study aims to develop and validate ML models using EHR and CGA data to predict discharge destinations of geriatric surgical inpatients. Our objective is to create an explainable and uncertainty-aware decision support tool that enhances individualized discharge planning, improves operational efficiency, and ultimately supports patient-centered outcomes in an aging surgical population.

## Methods

### Study population

The present study builds upon the previously published observational and AI development study of the SURGE-Ahead project [14]. The study population comprised geriatric surgical inpatients admitted to the trauma surgery, general and visceral surgery, and urology departments of Ulm University Medical Center between February 2023 and March 2024. We included patients aged 70 and older awaiting surgical intervention with an Identification of Seniors at Risk (ISAR) score of 2 or higher [20]. Exclusion criteria included a life expectancy of less than 3 months, inability to provide informed consent with lack of legal guardian, limited communication abilities, participation in other interventional studies, and anticipated postoperative hospital stays of less than 3 nights. Detailed recruitment procedures are described in the original publication [14] and visualized in the STROBE chart (Supplementary Figure S1) [21]. Of the 178 participants enrolled, 169 patients reached the primary endpoint and were available for the analysis.

### Explanatory variables

Patient data from a minimum geriatric data set (MGDS) [13] and information regarding the surgical procedure were considered, including age, sex, type of surgery (trauma, general/visceral surgery, urology), body mass index (BMI), Nutritional Risk Screening (NRS) [22], Montreal Cognitive Assessment 5 min protocol (MoCA 5-min) [23, 24], presence of dementia, Patient Health Questionnaire-4 (PHQ-4) [25], ISAR Screening [20], Clinical Frailty Scale (CFS) [26], American Society of Anesthesiologists score (ASA) [27], number of medications, care level (class I–V), nursing services received (none, at home, assisted living, nursing home), living situation (e.g., living alone), Barthel Index [28] measured at admission and on postoperative days 1 and 3, Charité Mobility Index (CHARMI) [29], New Mobility Score (NMS) [30], history of falls, emergency surgery status, time from admission to surgery, cut-to-suture time, length of stay in the intensive care unit, and overall length of hospital stay. German MoCA subtests were based on the validated German Version 8.1 of the full MoCA test. To ensure data quality, all data were collected by study nurses who were specifically trained for this study.

### Outcomes

In our previous analysis, we assessed the congruence between standard-of-care discharge decisions and geriatric expert recommendations [14], yielding a 73% overlap. However, in this study, we shifted the point of comparison: Rather than assessing congruence with the standard of care, we compared expert recommendations to suggestions generated by a ML algorithm, allowing us to evaluate the potential for AI in clinical decision support systems (CDSS). Standard-of-care discharge decisions refer to discharge destinations determined in routine practice, where geriatric consultation was provided according to existing referral practices rather than systematically to all eligible patients. Geriatric discharge destinations were categorized as home, acute geriatric care unit, post-acute rehabilitation facility, or nursing home, reflecting the southwest German regional care landscape [31]. Among the 169 patients included in the study, 104 were discharged home, 41 to an acute geriatric care unit, 5 to a rehabilitation facility, 17 to a nursing home, and 1 to a non-geriatric acute care hospital. In contrast, the expert recommendations were informed by in-person geriatric consultations, MGDS, follow-up data, and patient preferences, and were finalized through joint review by two experienced geriatricians. Of the 169 patients included in the study, 79 were recommended for discharge home, 75 for transfer to an acute geriatric care unit, 9 to a rehabilitation facility, and 6 to a nursing home. The expert recommendations are the ground truth for the present ML algorithm development and validation study. For further details, refer to the original publication of the observational and AI development study from the SURGE-Ahead project [14].

### Algorithm development and validation

For algorithm development, candidate features were pre-selected based on a recent scoping review conducted by our working group, identifying key predictors of appropriate discharge destinations [32], and supplemented with expert domain knowledge. Feature selection, model training, and evaluation were conducted within a fivefold cross-validation (CV) framework, with all imputation, fitting, tuning, and calibration procedures confined to the training data. To address the limited sample size, the training set was augmented with additional records from an orthogeriatric trauma registry (the so-called “ATZ registry”; *n* = 82 samples; assessed by geriatric liaison service). This process was guided by a companion study [33], which was explicitly designed to determine a suitable method for this data augmentation. To minimize the risk of information leakage, we reused the folds established in that study. First, feature selection was performed using single-step recursive feature elimination based on Gini impurity reduction in 1000 bootstrapped decision trees. Features selected across folds were aggregated into a single set. Inclusion of the orthogeriatric trauma registry samples during training was treated as a hyperparameter (see below). Then, missing data in the training set were imputed using variable-specific mean, median, or mode imputation. As the classification algorithm, adaptive boosting with decision tree stumps (AdaBoost [34]) was implemented. Decision tree stumps are essentially binarized features that act as simple decision rules. AdaBoost combines these rules into an ensemble, where each additional rule is specifically designed to correct the classification errors made by the previous ones. The selection of AdaBoost over more complex,’black-box’ architectures was a deliberate choice to prioritize model transparency. In a clinical setting, particularly regarding discharge planning for frail older adults, the ability to trace a prediction back to specific clinical thresholds is more valuable than marginal gains in accuracy. This approach ensures that the AI remains a supportive tool that clinicians can critically evaluate and, if necessary, override based on their professional judgment. Multiclass classification was implemented using a one-vs-one strategy, resulting in a classifier for each possible combination. This strategy is advantageous when encountering class imbalance and improves interpretability [35]. Within each CV fold, hyperparameters of the AdaBoost classifier were optimized using a low-dimensional grid search (see below), and sigmoid calibration was applied using an internal CV within the training fold [36, 37]. Hyperparameters considered were inclusion versus exclusion of the additional ATZ registry data (Yes/No), number of AdaBoost estimators (1 to 20, step size 1), AdaBoost learning rate (0.5 to 1.0 in increments of 0.1), and number of splits for sigmoid calibration (2 to 5, step size 1). Model performance was evaluated on the held-out test folds in each CV iteration for all hyperparameter configurations in the grid, and the test set predictions of the best-performing configuration across the grid were retained for statistical analysis. For model inspection and as a possible candidate for deployment in the SURGE-Ahead project, a single final model was trained on the full (non-CV) dataset using the hyperparameters that achieved the best performance. An overview of algorithm development, including data flow and model architecture, is provided in Supplementary Figures S2 and S3. To ensure the robustness of this choice, a comparison with TabPFN 3 [38] and a Random Forest [39] model was conducted (see Supplementary Methods and Results), which did not demonstrate superior performance. All modeling was performed using Python 3.12.2 and scikit-learn 1.7.1, with the full codebase publicly available on GitHub at https://github.com/IfGF-UUlm/SA_COC. Model development and reporting adhered to the TRIPOD + AI framework (Supplementary Table S1) [40].

### Statistical analysis

The test set predictions from the best-performing model in the CV process were saved for statistical analyses. Model performance with respect to expert-recommended discharge destinations was assessed using accuracy (primary outcome) and receiver operating characteristic (ROC). Accuracy is defined as the proportion of total correct predictions, including both true positives and true negatives, relative to the total number of cases analyzed. To further enhance the clinical translation of the model’s performance, we calculated positive predictive values (PPV) and negative predictive values (NPV) for each discharge destination. Additionally, a confusion matrix was generated to visualize the distribution of true positives, true negatives, and misclassifications. A confusion matrix is a technical tool used to visualize the performance of an algorithm by comparing predicted classifications against actual observed outcomes. Model calibration was evaluated using the Brier score and calibration curves. The 95% confidence intervals (CI) for all metrics were calculated using bootstrapping with 1000 iterations. To mimic the planned human-in-the-loop approach in the SURGE-Ahead project, we additionally analyzed performance after removing the 15% of predictions with the lowest predicted class probabilities (highest uncertainty), as these outputs would be reviewed by a geriatrician as part of the planned SURGE-Ahead interventional trial. This 15% uncertainty threshold was defined a priori based on clinical judgement. Additional performance metrics were then calculated for the remaining 85% of the most confident predictions. For both approaches, the primary endpoint was compared to the previously published standard-of-care results [14], using the McNemar’s test. Patient-level linkage across datasets was reconstructed using a deterministic matching procedure based on unique combinations of participant characteristics; 167 of 169 participants could be uniquely matched. Two participants could not be reliably matched due to protocol-related modifications of dropout criteria and were excluded from the paired analysis. Across all patients, explanatory variables were analyzed by comparing groups with congruent (prediction matched expert recommendation) and incongruent discharge decisions, mirroring the analytical approach in our previous research on standard-of-care decisions [14]. Descriptive statistics were calculated to characterize the study population, stratifying by discharge destination congruence. Continuous variables are presented as means and standard deviations, while frequencies and percentages are reported for binary variables. Exploratory analyses were conducted to compare groups using Student’s t-tests or Mann–Whitney U tests for continuous or discrete variables (depending on normality), the Mann–Whitney U test for ordinal variables, and Chi-square tests with Yates’ continuity correction for binary variables. All analyses were performed using the scipy 1.12 and statsmodels 0.14 libraries for Python, with a significance level of *p* < 0.05. As an exploratory analysis, no correction for multiple comparisons was applied.

## Results

### Study population

The study population consisted of 169 older patients with a mean age of 80.5 years (SD ± 6.3), comprising 68 (41%) males and 101 (59%) females. The majority of patients underwent trauma surgery (76%). The cohort was characterized by significant geriatric vulnerabilities, including a mean ISAR score of 3.0 (SD ± 1.0), a mean Barthel Index of 86.0 (SD ± 19.2), and a moderate medication burden averaging 8.9 medications (SD ± 3.8). Most patients had a history of falls, and the average hospital stay was 12.5 days (SD ± 10.3). Based on the geriatric expert evaluations, 79 patients were recommended for discharge home, 75 for transfer to an acute geriatric care unit, 9 to a rehabilitation facility, and 6 to a nursing home. The congruence of standard-of-care with expert-recommended discharge decisions was 73%. A detailed description of the study population characteristics has been previously reported by Leinert and colleagues [14]. Note that datasets slightly changed between studies, because of protocol-related modifications of dropout criteria, affecting two cases (1 in, 1 out).

### Model performance and statistical analysis

**Fig. 1:**
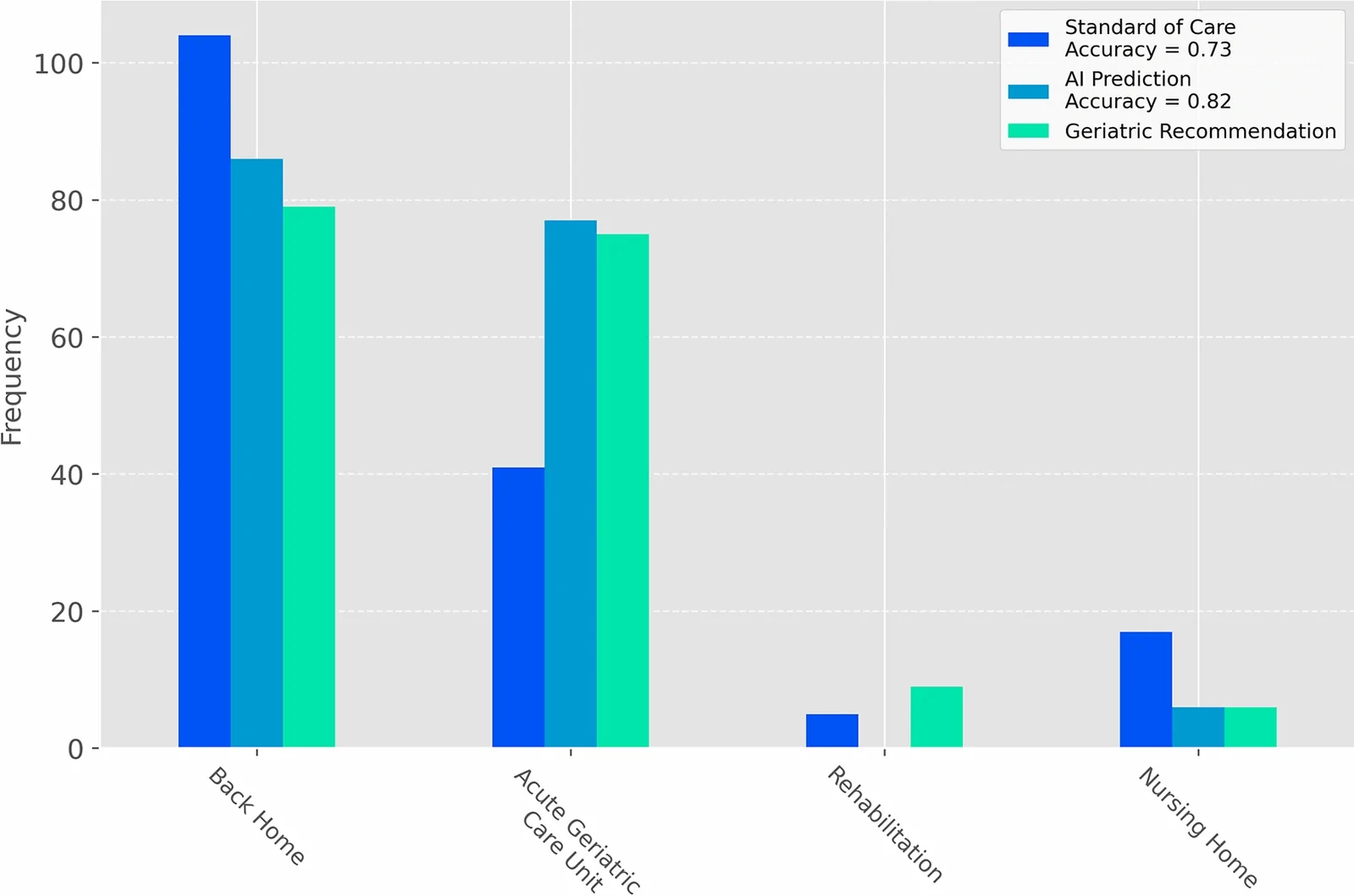
Primary outcome. The bar charts compare discharge decisions between the standard of care, artificial intelligence (AI) predictions, and geriatric expert recommendations. The AI improved alignment with geriatric expert recommendations from 73 to 82% (*p* = 0.041) and produced overall better-fitting distributions. Rehabilitation = post-acute rehabilitation facility

We first evaluated the overall predictive performance of the ML model in reproducing the expert-recommended discharge destination (Table 1). The model achieved an internal validation accuracy of 0.82 [95% CI 0.76–0.86], indicating that in 82% of cases, the AI prediction matched the geriatric experts’ recommendation (Fig. 1). The clinical utility of the ML model is further detailed in the confusion matrix (Table 2). The model demonstrated PPV of 0.84 for discharge home, 0.81 for acute geriatric care unit, and 0.67 for nursing home; PPV for rehabilitation was not estimable because the model did not select rehabilitation as the top prediction in any case, sensitivity for this class was 0/9. The NPV were 0.92 for discharge home, 0.86 for acute geriatric care unit, 0.95 for rehabilitation facility, and 0.99 for nursing home. Compared to the standard-of-care decisions, the ML algorithm yielded a relative increase of 11% in correct discharge recommendations (138 vs. 124; 82% vs. 73% accuracy), suggesting that the AI-supported approach could potentially reduce the rate of suboptimal discharge decisions in routine clinical practice.

**Table 1:** Study population characteristics

	All (*n* = 169)		AI congruent (*n* = 138)		AI incongruent (*n* = 31)		Group Difference
	Mean (± SD)	*n* (%)	Mean (± SD)	*n* (%)	Mean (± SD)	*n* (%)	*p*-value
Age (years)	80.5 (± 6.3)		80.6 (± 6.5)		80.1 (± 5.3)		0.652
Sex (male)		68 (41%)		58 (42%)		10 (33%)	0.482
Trauma surgery (yes/no)		127 (76%)		104 (76%)		23 (77%)	> 0.999
General and visceral surgery (yes/no)		22 (13%)		17 (12%)		5 (17%)	0.744
Urology (yes/no)		18 (10%)		16 (12%)		2 (7%)	0.634
BMI (kg/m^2^)	26.3 (± 5.0)		26.3 (± 4.9)		26.2 (± 5.9)		0.700
NRS (yes/no)		78 (46%)		66 (48%)		12 (40%)	0.620
MoCA 5 min (score)	21.6 (± 5.9)		21.6 (± 5.8)		21.5 (± 6.4)		0.908
Dementia (yes/no)		19 (11%)		15 (11%)		4 (13%)	0.956
PHQ-4 (score)	2.5 (± 2.7)		2.3 (± 2.6)		3.5 (± 3.2)		0.055
ISAR (score)	3.0 (± 1.0)		3.0 (± 0.9)		3.4 (± 1.2)		0.087
CFS (score)	4.2 (± 1.8)		4.1 (± 1.8)		4.6 (± 1.6)		0.163
ASA (class)	2.9 (± 0.5)		2.9 (± 0.5)		3.1 (± 0.4)		**0.019**
Number of medications (n)	8.9 (± 3.8)		8.5 (± 3.5)		10.6 (± 4.8)		**0.005**
Care level (class)	1.0 (± 1.3)		1.0 (± 1.3)		1.0 (± 1.2)		0.785
Nursing services (class)	1.1 (± 0.8)		1.1 (± 0.7)		1.2 (± 0.8)		0.601
Living alone (yes/no)		77 (46%)		59 (43%)		18 (60%)	0.138
Barthel Index (score)	86.0 (± 19.2)		85.8 (± 19.3)		86.7 (± 19.3)		0.900
CHARMI (score)	7.9 (± 2.7)		7.9 (± 2.7)		7.9 (± 2.7)		0.918
NMS (score)	6.3 (± 2.5)		6.3 (± 2.6)		6.1 (± 2.3)		0.534
Falls (yes/no)		104 (62%)		84 (61%)		20 (67%)	0.959
Emergency OP (yes/no)		95 (57%)		73 (53%)		22 (73%)	0.071
Time to OP (h)	63.1 (± 88.1)		57.8 (± 88.0)		86.7 (± 85.7)		**0.034**
Cut-to-suture time (min)	88.9 (± 67.1)		87.8 (± 62.9)		94.0 (± 84.8)		0.859
Length of stay ICU (min)	402.4 (± 979.8)		316.0 (± 523.5)		775.6 (± 1966.4)		0.181
Length of stay (days)	12.5 (± 10.3)		12.3 (± 11.0)		13.4 (± 6.6)		**0.038**

Significant differences are highlighted. *BMI *body mass index, *NRS* nutritional risk screening, *MoCA* montreal cognitive assessment, *PHQ-4* patient health questionnaire-4, *ISAR* identification of seniors at risk, *CFS* clinical frailty scale, *ASA* American Society of Anesthesiologists score, *CHARMI* Charité mobility index, *NMS* new mobility score, *ICU* intensive care unit, *OP* operation

**Table 2:** Confusion matrix showing the distribution of AI-predicted discharge destinations versus expert-recommended ground truth

AI prediction	Home	Acute geriatric care unit	Rehabilitation	Nursing home
Ground truth				
Home	72	7	0	0
Acute geriatric care unit	11	62	0	2
Rehabilitation	3	6	0	0
Nursing home	0	2	0	4

The cells contain absolute numbers; the diagonal (top left to bottom right; shaded) represents correct predictions, while off-diagonal cells illustrate specific misclassifications between the different care levels

**Fig. 2:**
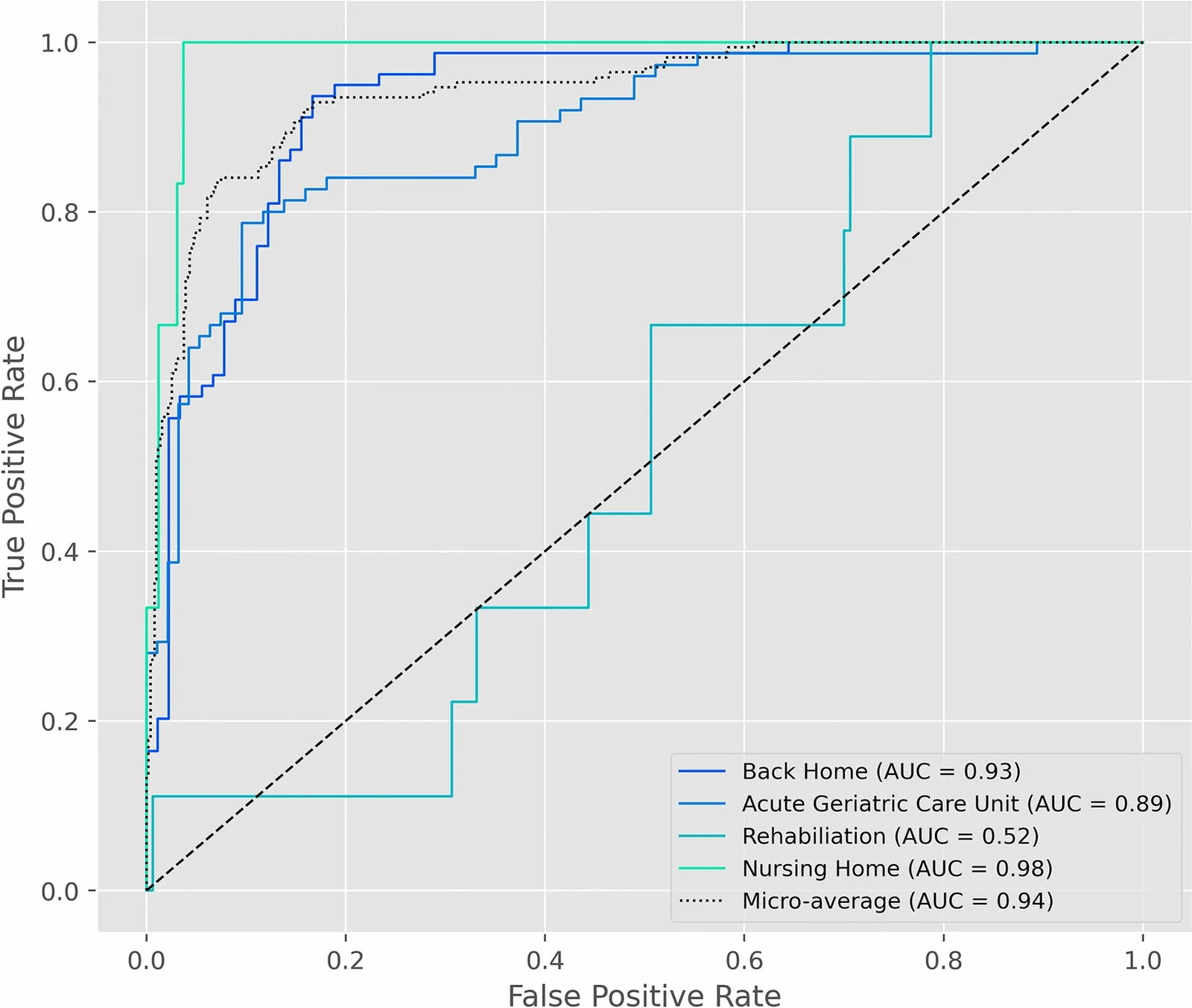
Receiver operating characteristic (ROC) curves. The micro-averaged area under the curve (AUC) is 0.94, indicating excellent overall discrimination. The major discharge destinations, ‘home’ and ‘acute geriatric care unit’, yield AUCs of 0.93 and 0.89, respectively. ‘Rehabilitation’ is an outlier, with an AUC of 0.52, highlighting challenges in accurately classifying this discharge destination

**Fig. 3:**
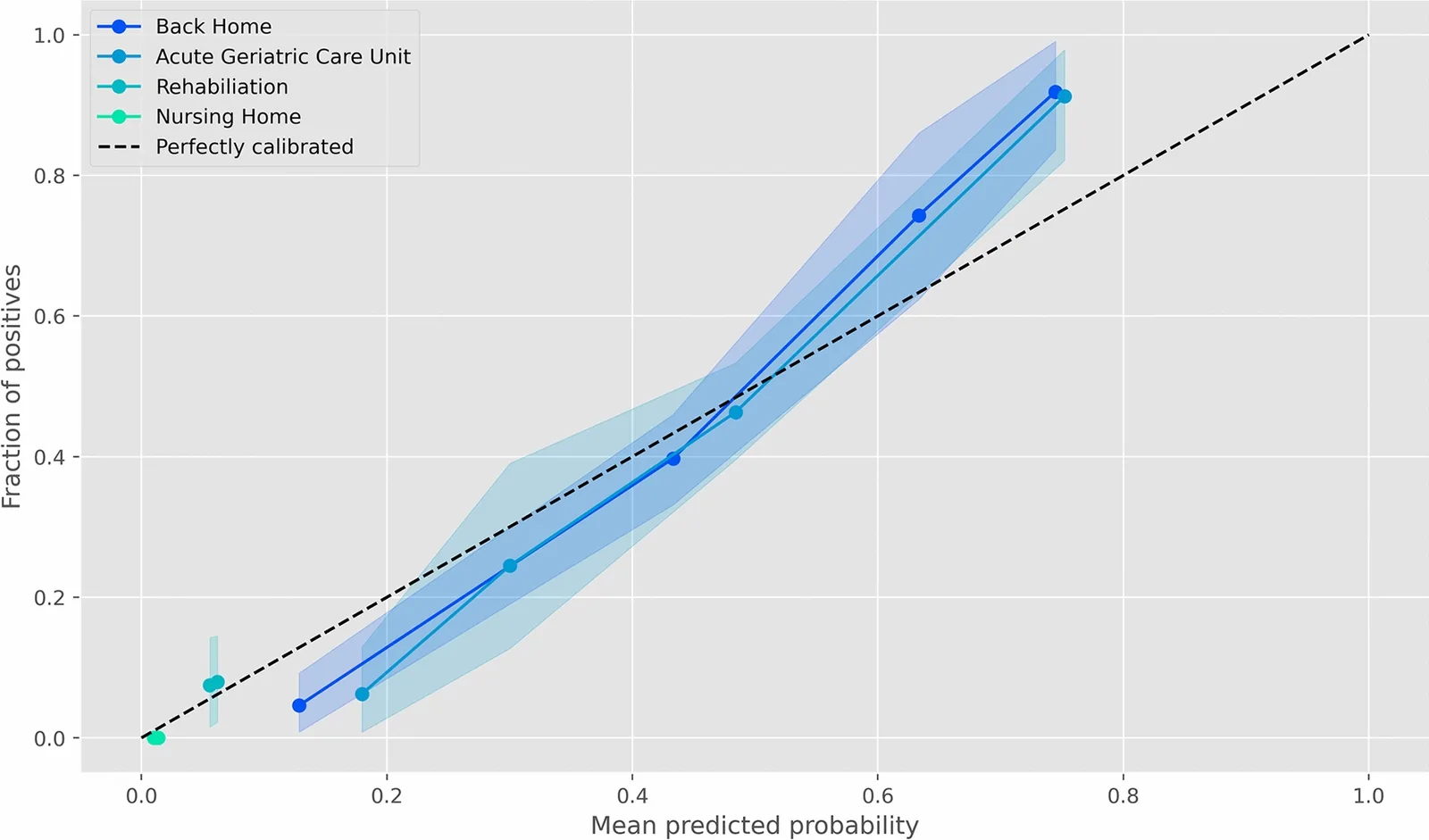
Calibration curves. Calibration plots showing the relationship between predicted probabilities and observed frequencies for the four discharge destinations: home, acute geriatric care unit, rehabilitation, and nursing home. Shaded areas indicate the 95% confidence intervals. Overall, predicted and observed probabilities show close agreement

To further assess the model’s ability to discriminate among the different care levels, we analyzed the ROC curve. The micro-averaged AUC was 0.94 [95% CI 0.92–0.96], indicating high overall discriminative power. When looking at specific destinations (Fig. 2), the model was accurate in identifying patients suitable for discharge home (AUC 0.93), admission to an acute geriatric care unit (AUC 0.89), and nursing home placement (AUC 0.98), whereas predicting the need for a rehabilitation facility remained challenging (AUC 0.52). The reliability of probability estimates was confirmed by the Brier Score (0.33 [95% CI 0.30–0.37]) and calibration curves, which show that the predicted probabilities closely align with the observed outcomes (Fig. 3). To simulate a clinical implementation, we analyzed the model’s performance using a human-in-the-loop approach. In this scenario, the 15% of cases with the highest prediction uncertainty would be flagged for manual review by a geriatrician. For the remaining 85% of confident predictions, the accuracy increased to 0.85 [95% CI 0.80–0.90] and the micro-averaged ROC AUC rose to 0.95 [95% CI 0.93–0.97]. This demonstrates that the model can handle most cases while identifying complex patients who require expert human intervention.

### Model inspection

We then investigated which clinical factors most strongly influenced the model’s decisions, specifically focusing on the distinction between discharging a patient home versus transferring them to an acute geriatric care unit. The most influential predictors identified by the model were a Barthel Index of 50 or less on postoperative day 3, a Clinical Frailty Scale (CFS) score of 5 or higher, and a Barthel Index of 60 or less on postoperative day 1. Other key drivers included age (88 years or older), admission Barthel Index (65 or less), and polypharmacy (8 or more medications). Based on the model inspection, we derived a preliminary scoring system to assist clinicians in identifying patients who may benefit from an acute geriatric care unit. This system assigns points based on the most influential predictors: 3 points for a Barthel Index ≤ 50 on postoperative day 3, and 1 point each for a Barthel Index ≤ 65 at admission, a Clinical Frailty Scale (CFS) score ≥ 5, polypharmacy (≥ 8 medications), and age ≥ 88 years (Table 2). Using a Youden-optimized cutoff of ≥ 4, the scoring system achieved a sensitivity of 0.85 and a specificity of 0.86. It should be noted that these performance estimates were calculated using the training data and may therefore be overconfident.

### Impact on clinical decision-making

Finally, we compared patient characteristics based on whether the AI prediction was congruent or incongruent with the expert recommendation (Table 1). Patients with incongruent predictions are those for whom the AI suggested a different discharge destination than the one recommended by the geriatricians. The AI algorithm demonstrated significantly improved congruence with expert recommendations compared to previously published standard-of-care decisions (*p* = 0.041). Specifically, the ML model correctly predicted the discharge destination for 138 of 169 patients (82%), compared to 124 of 169 patients (73%) in the standard-of-care group, representing 15 additional correct discharge recommendations. This improvement was further enhanced in the human-in-the-loop approach, where the accuracy for the most confident predictions rose to 85% (*p* = 0.006).

Between expert-congruent and -incongruent predictions, there were no statistically significant differences in age, sex, or surgical specialty. However, patients with incongruent predictions had a statistically significant higher ASA classification (3.1 ± 0.4 vs. 2.9 ± 0.5) and took more concomitant medications (10.6 ± 4.8 vs. 8.5 ± 3.5). Additionally, the time to operation was longer in the incongruent group (86.7 ± 85.7 h vs. 57.8 ± 88.0 h) as was the length of hospital stay (13.4 ± 6.6 days vs. 12.3 ± 11.0 days).

## Discussion

The present algorithm development and internal validation study within the SURGE-Ahead project demonstrates the high predictive performance of AI-based tools for discharge planning in older adults following surgery. Rather than demonstrating practical feasibility in a real-world clinical workflow, this study confirms the model’s internal validity and discriminative power. Compared with the standard of care, the congruence with expert recommendations increased from 73 to 82% (*p* = 0.041), establishing an evidence base for the potential of AI to support clinical decision-making. The PPV and NPV for discharge home (PPV 0.84, NPV 0.92) and acute geriatric care unit (PPV 0.81, NPV 0.86) underscore the potential of the tool in a clinical setting. By increasing the proportion of correct discharge recommendations compared to the standard of care, the ML model could support surgeons in the absence of geriatricians by suggesting potentially more suitable alternatives, thereby helping to minimize the risk of premature discharge home or unnecessary institutionalization.

It is important to note that our model was specifically designed to output calibrated probabilities rather than discrete labels. This decision was driven by the need to convey predictive uncertainty, which is critical for patient safety and aligns with the requirements of Medical Device Regulation (MDR) compliant applications in healthcare. By avoiding a rigid labeling system, we prevent overreliance on a single AI-generated decision. Instead, we plan to implement a’human-in-the-loop’ strategy: cases characterized by high uncertainty, where the model cannot provide a high-probability recommendation, are automatically flagged for expert review by a geriatrician. This approach ensures that the AI serves as a supportive tool rather than a replacement for clinical judgment, prioritizing patient welfare by ensuring that complex or ambiguous cases are always managed by human expertise. Our ML algorithm demonstrated good calibration indicating that the empirical probabilities closely aligned with the predicted probabilities. Moreover, a monotonic relationship was observed between the degree of certainty and the accuracy of the proposed discharge destination (Supplementary Figure S4), suggesting that the model’s confidence scores provide meaningful guidance for clinical use. Building on this, we explored a human-in-the-loop approach, where the 15% of predictions with the lowest certainty trigger a geriatric consultation. Using this strategy, the congruence of the remaining AI predictions increased to 85%, representing an improvement over the standard of care. By leveraging predicted probabilities to identify cases requiring expert input, this approach enhances patient safety and underscores the value of uncertainty quantification in safety–critical healthcare applications. Furthermore, opting for an intrinsically explainable model renders AI decisions auditable by physicians, which can foster trust in AI systems and facilitate the identification of inaccuracies. This empowers physicians to deviate from AI suggestions when clinically indicated and aligns with ethical objectives [41, 42].

Model inspection revealed that the most important predictors for optimal discharge decisions were a Barthel Index ≤ 50 at postoperative day 3 and a CFS ≥ 5. These thresholds are particularly noteworthy, as they emerged solely from the data, without human input, yet replicated established thresholds for clinical decision-making: a Barthel Index ≤ 50 generally indicates the need for continued inpatient treatment, whereas a CFS of 5 or higher corresponds to the established threshold for a frailty syndrome. In general, the predictors for discharge to an acute geriatric care unit can be grouped into assessments of activities of daily living and geriatric hallmarks, providing a comprehensive overview of patients who may benefit from geriatric treatment. Based on these insights, we developed an additional scoring system to aid in determining patient eligibility for acute geriatric care (Table 3), facilitating easy implementation.

**Table 3:** Simplified scoring system predicting the suitability for discharge to an acute geriatric care unit

Patient characteristic	Points
*Activities of daily living*	
Barthel Index at admission (score) ≤ 65	1
Barthel Index on postoperative day 3 (score) ≤ 50	3
*Geriatric hallmarks*	
Clinical Frailty Scale (CFS; score) ≥ 5	1
Number of medications (*n*) ≥ 8	1
Age (years) ≥ 88	1

The Youden-optimized cutoff is ≥ 4, with a sensitivity of 0.85 and a specificity of 0.86. Note that the performance was tested on the training data, so estimates may be overconfident

Compared to previous studies predicting discharge destination or timing, our model demonstrated significantly improved predictive performance. Specifically, Morris and colleagues (2022) predicted discharge within the next 24 h with an ROC AUC of 0.87, while Wei and colleagues (2024) achieved an ROC AUC of 0.73 for discharge destination prediction [16, 17]. In contrast, the micro-averaged ROC AUC across all discharge categories in our study was 0.94, with destination-specific ROC AUC values around 0.90 for the major discharge destinations. This represents a substantial improvement in discrimination, potentially reflecting the added value of incorporating comprehensive geriatric assessment-based variables into the model.

Given the limited sample size and the nature of our internal validation, the results of this study should be interpreted as a proof-of-concept analysis. We have demonstrated that an AI-driven approach can effectively mirror expert geriatric recommendations and outperform the standard of care in a controlled dataset. However, this does not yet constitute a validation of clinical efficacy. The transition from a high-performing algorithm to a reliable clinical tool requires evidence from clinical trials. Therefore, our current findings serve as the necessary empirical foundation for our ongoing interventional study, which aims to determine whether this decision support actually improves patient outcomes and operational efficiency in routine geriatric care.

## Limitations

A significant limitation of this study is the small number of observations in the ‘rehabilitation facility’ (*n* = 9) and ‘nursing home’ (*n* = 6) classes. With such small sample sizes, establishing stable decision boundaries and achieving reliable probability calibration is inherently challenging. To mitigate these constraints, we implemented several strategies: first, we utilized a one-vs-one classification strategy, which derives the final prediction from an ensemble of six binary classifiers rather than a single one-vs-all model. Second, we employed a cross-validated calibration approach, utilizing an inner n-fold cross-validation loop within each training fold to generate more robust calibration data. Despite these measures, the predictive stability varied by class. While patients destined for nursing homes remained largely separable, as indicated by the ROC analysis, the prediction of rehabilitation placement was less stable. We therefore think that the model’s performance for these minority classes should be interpreted with caution. Examination of the 9 patients recommended for a rehabilitation facility revealed that 3 were assigned to this destination solely due to bed availability and would otherwise likely have been discharged home to await rehabilitation. In 5 cases, concerns regarding post-discharge care prompted experts to recommend extending inpatient stay until a rehabilitation placement became available; these patients likely would have been recommended to be discharged home or to an acute geriatric care unit. In conclusion, discharge to rehabilitation facilities is largely influenced by situational factors and cannot be reliably predicted from EHR data. Therefore, AI should not dictate these decisions, which should be guided by local healthcare structures. Nevertheless, this limitation does not affect the model’s validity for other decisions. Furthermore, we acknowledge a potential for optimistic estimation of model performance. Although model performance was assessed using a cross-validation framework, the hyperparameters were optimized within this same process, and no entirely independent holdout test set was utilized due to the limited sample size. While we employed a cautious approach by limiting the hyperparameter grid to reduce the risk of overfitting, these results should be interpreted as internal validation estimates. Consequently, the actual performance in an external, real-world setting may be lower. While the results of this study demonstrate that the algorithm can accurately mirror geriatric expert recommendations, the practical feasibility, operational integration, and actual impact on patient outcomes remain to be determined. To this end, the proposed AI algorithm is now being evaluated in an ongoing intervention trial to assess its real-world feasibility and its ability to improve discharge quality. Finally, we acknowledge that the 15% uncertainty threshold used in our human-in-the-loop analysis was selected for demonstration purposes and is not derived from an independently optimized probability cutoff. While the standard for establishing such a threshold would require a separate validation dataset, the limited sample size made this approach unfeasible without significantly compromising model training. Consequently, the optimal threshold for triggering manual expert review remains to be determined. This limitation will be addressed in our ongoing interventional trial, which will allow us to refine the uncertainty thresholds using an external validation cohort and evaluate the practical impact of different manual review triggers on discharge quality.

## Conclusion

This study describes the development and internal validation of an ML algorithm to support discharge planning for geriatric surgical patients. It shows that an AdaBoost-based machine learning model can improve alignment with geriatric expert discharge recommendations compared with standard-of-care discharge planning. The model achieved 82% congruence with expert recommendations, increasing to 85% with a human-in-the-loop approach, representing a statistically significant improvement over the standard of care. Although these results should be interpreted as proof-of-concept, they provide the necessary foundation for its evaluation in an ongoing interventional trial.

## Supplementary Information

Below is the link to the electronic supplementary material.

Supplementary file1 (DOCX 632 KB)

## Data Availability

Data used in this specific analysis can also be accessed on the project’s GitHub page: https://github.com/IfGF-UUlm/SA_COC

## Abbreviations

CGA: Comprehensive geriatric assessment
AI: Artificial intelligence
ML: Machine learning
EHR: Electronic health record
ISAR: Identification of seniors at risk
MGDS: Minimum geriatric data set
BMI: Body mass index
NRS: Nutritional risk screening
MoCA 5-min: Montreal Cognitive Assessment 5-min protocol
PHQ-4: Patient Health Questionnaire-4
CFS: Clinical Frailty Scale
ASA: American Society of Anesthesiologists score
CHARMI: Charité Mobility Index
NMS: New Mobility Score
CDSS: Clinical decision support systems
TRIPOD: Transparent reporting of a multivariable prediction model for individual prognosis or diagnosis
CV: Cross-validation
AdaBoost: Adaptive boosting
ROC: Receiver operating characteristic
CI: Confidence intervals
AUC: Area under the curve
STROBE: STrengthening the Reporting of OBservational studies in Epidemiology
TabPFN: Tabular Prior-data Fitted Network
ICU: Intensive care unit
OP: Operation
