# Supplementary material for: Standard-of-care vs. machine learning-recommended discharge destinations for geriatric surgical inpatients: algorithm development and internal validation
Source: Eur Geriatr Med. 2026 Jul 31;17(5):3077–88. doi: 10.1007/s41999-026-01566-z (PMC13755074; doi:10.1007/s41999-026-01566-z)
Supplement: Supplementary file 1 [file 41999_2026_1566_MOESM1_ESM.docx]

Standard-of-Care vs. Machine Learning–Recommended Discharge Destinations for Geriatric Surgical Inpatients: Algorithm Development and Internal Validation

Supplementary Material

Thomas Derya Kocar^1^, Utz Lovis Rieger^2^, Filippo Maria Verri^1^, Pauline Maier^1^, Christoph Leinert^1^, Simone Brefka^1^, Christian Bolenz^3^, Nuh Rahbari^4^, Florian Gebhard^5^, Dhayana Dallmeier^1,6^, Michael Denkinger^1^, Hans Kestler^2^

^1^ Institute for Geriatric Research Ulm, Ulm University Medical Center, Ulm, Germany

^2^ Institute of Medical Systems Biology, Ulm University, Ulm, Germany

^3^ Department of Urology and Pediatric Urology, Ulm University Medical Center, Ulm, Germany.

^4^ Department of General and Visceral Surgery, Ulm University Medical Center, Ulm, Germany.

^5^ Department of Orthopedic Trauma, Hand, Plastic and Reconstruction Surgery, Ulm University Medical Center, Ulm, Germany

^6^ Department of Epidemiology, Boston University School of Public Health, Boston, MA, United States

# Supplementary Material

## Supplementary Methods

The model was implemented as a cross-sectional analysis, treating measurements taken at different time points (such as the Barthel Index at admission and on postoperative days 1 and 3) as separate, independent predictors rather than as a time series. To evaluate the robustness of the AdaBoost model, two additional machine learning architectures were implemented and compared: a Random Forest (RF) classifier and TabPFN. The Random Forest is an ensemble learning method that constructs a multitude of decision trees during training and outputs the mode of the classes for classification, thereby reducing the risk of overfitting compared to single decision trees. TabPFN was utilized as a state-of-the-art transformer-based model specifically pre-trained on a vast number of synthetic tabular datasets, allowing it to perform predictions in a few-shot manner without traditional iterative training.

Both models were integrated into the same 5-fold cross-validation (CV) framework described in the main manuscript. For each fold, the training set was augmented with data from the orthogeriatric trauma registry (ATZ), and missing values were handled using variable-specific mean, median, or mode imputation derived solely from the training data to prevent data leakage. Both classifiers were implemented using their default hyperparameters with a fixed random seed (42) to ensure reproducibility. The models treated measurements at different time points as independent predictors, consistent with the primary analysis. Model performance was evaluated based on the accuracy of the predictions on the held-out test folds.

## Supplementary Results

The comparison of the classification algorithms demonstrated that neither the Random Forest nor the TabPFN model provided a significant performance advantage over the primary AdaBoost approach. The TabPFN classifier achieved an overall cross-validated accuracy of 0.78, while the Random Forest classifier reached an accuracy of 0.80. These results confirm that the choice of AdaBoost, which prioritizes clinical transparency and interpretability over higher architectural complexity, was appropriate for the clinical context of discharge planning in frail older adults.

## Supplementary Figures and Tables

### Supplementary Figures

**Supplementary Figure S1: STROBE Chart.** From an initial cohort of 1291 patients assessed for eligibility, 169 patients were analyzed for the primary endpoint. This chart is reproduced from the original publication of the SURGE-Ahead observational trial [1]. STROBE = STrengthening the Reporting of OBservational studies in Epidemiology, ISAR = Identification of Seniors at Risk

**Supplementary Figure S2: Data Flow Chart.** Model training was conducted both with and without additional data from the orthogeriatric trauma registry (ATZ register), with the inclusion yielding better performance.

**Supplementary Figure S3: Model Architecture.** The classification framework is based on an AdaBoost ensemble of decision tree stumps trained using the SAMME (Stagewise Additive Modeling using a Multi-class Exponential loss function) algorithm. To accommodate the four discharge destinations, a one-vs-one strategy was employed, resulting in six pairwise binary classifiers. For each prediction, every binary classifier contributes a vote to its predicted class and a pairwise confidence score. The multiclass decision function is computed as the sum of class votes and the normalized sum of pairwise confidence scores across all six classifiers, yielding a continuous score for each discharge destination. The resulting multiclass decision scores are subsequently calibrated using sigmoid calibration to obtain a probability vector. To enhance explainability, we inspected the AdaBoost weak learners and their estimator weights regarding the clinically relevant differentiation between ‘back home’ and ‘acute geriatric care’. During deployment within the SURGE-Ahead project dashboard, missing predictor values are handled using mean, median, or mode imputation derived from the training dataset.

**Supplementary Figure S4: Probability thresholds versus accuracy.** The chart shows a monotonic relationship between probability thresholds (cut-off) and accuracy.

**Supplementary Figure S5: Receiver Operating Characteristic (ROC) curve for the scoring system.** The scoring system predicts the suitability for discharge to an acute geriatric care unit. The Youden-optimized cut-off is ≥ 4, with a sensitivity of 0.85 and a specificity of 0.86. Note that the performance was tested on the training data, so estimates may be overconfident.

### Supplementary Tables

**Supplementary Table S1: TRIPOD+AI Checklist.** Pages refer to the original submission and may have changed in editorial post-production. TRIPOD = Transparent Reporting of a multivariable prediction model for Individual Prognosis Or Diagnosis, n/a = not applicable.

| **Section/Topic** | **Checklist Item** | **Reported on page(s)** |
| --- | --- | --- |
| **Title** | | |
| *Title* | Identify the study as developing or evaluating the performance of a multivariable prediction model, the target population, and the outcome to be predicted. | 1 |
| **Abstract** | | |
| *Abstract* | See TRIPOD+AI for Abstracts checklist. | 2 |
| **Introduction** | | |
| *Background* | Explain the healthcare context (including whether diagnostic or prognostic) and rationale for developing or evaluating the prediction model, including references to existing models. | 4, 5 |
|  | Describe the target population and the intended purpose of the prediction model in the context of the care pathway, including its intended users (e.g., healthcare professionals, patients, public. | 4, 5 |
|  | Describe any known health inequalities between sociodemographic groups. | 4 |
| *Objectives* | Specify the study objectives, including whether the study describes the development or validation of a prediction model (or both). | 5 |
| **Methods** | | |
| *Data* | Describe the sources of data separately for the development and evaluation datasets (e.g., randomised trial, cohort, routine care or registry data), the rationale for using these data, and representativeness of the data. | 5-8, Supplementary Figure S2 |
|  | Specify the dates of the collected participant data, including start and end of participant accrual; and, if applicable, end of follow-up. | 5, 6, Supplementary Figure S2 |
| *Participants* | Specify key elements of the study setting (e.g., primary care, secondary care, general population) including the number and location of centres. | 5, 6 |
|  | Describe the eligibility criteria for study participants. | 5, 6 |
|  | Give details of any treatments received, and how they were handled during model development or evaluation, if relevant. | n/a |
| *Data preparation* | Describe any data pre-processing and quality checking, including whether this was similar across relevant sociodemographic groups. | 6, 7 |
| *Outcome* | Clearly define the outcome that is being predicted and the time horizon, including how and when assessed, the rationale for choosing this outcome, and whether the method of outcome assessment is consistent across sociodemographic groups. | 6, 7 |
|  | If outcome assessment requires subjective interpretation, describe the qualifications and demographic characteristics of the outcome assessors. | 6, 7 |
|  | Report any actions to blind assessment of the outcome to be predicted. | n/a |
| *Predictors* | Describe the choice of initial predictors (e.g., literature, previous models, all available predictors) and any pre-selection of predictors before model building. | 7, Supplementary Figure S2 |
|  | Clearly define all predictors, including how and when they were measured (and any actions to blind assessment of predictors for the outcome and other predictors). | 6 |
|  | If predictor measurement requires subjective interpretation, describe the qualifications and demographic characteristics of the predictor assessors. | 6 |
| *Sample size* | Explain how the study size was arrived at (separately for development and evaluation), and justify that the study size was sufficient to answer the research question. Include details of any sample size calculation. | 5, 7, Supplementary Figure S1 |
| *Missing Data* | Describe how missing data were handled. Provide reasons for omitting any data. | 7 |
| *Analytical Methods* | Describe how the data were used (e.g., for development and evaluation of model performance) in the analysis, including whether the data were partitioned, considering any sample size requirements. | 8, Supplementary Figure S2 |
|  | Depending on the type of model, describe how predictors were handled in the analyses (functional form, rescaling, transformation, or any standardisation). | 8 |
|  | Specify the type of model, rationale, all model-building steps, including any hyperparameter tuning, and method for internal validation. | 8 |
|  | Describe if and how any heterogeneity in estimates of model parameter values and model performance was handled and quantified across clusters (e.g., hospitals, countries). See TRIPOD-Cluster for additional considerations. | n/a |
|  | Specify all measures and plots used (and their rationale) to evaluate model performance (e.g., discrimination, calibration, clinical utility) and, if relevant, to compare multiple models. | 9, 10 |
|  | Describe any model updating (e.g., recalibration) arising from the model evaluation, either overall or for particular sociodemographic groups or settings. | n/a |
|  | For model evaluation, describe how the model predictions were calculated (e.g., formula, code, object, application programming interface). | 7 |
| *Class Imbalance* | If class imbalance methods were used, state why and how this was done, and any subsequent methods to recalibrate the model or the model predictions. | 7 |
| *Fairness* | Describe any approaches that were used to address model fairness and their rationale. | n/a |
| *Model output* | Specify the output of the prediction model (e.g., probabilities, classification). Provide details and rationale for any classification and how the thresholds were identified. | 7, 8 |
| *Training versus evaluation* | Identify any differences between the development and evaluation data in healthcare setting, eligibility criteria, outcome, and predictors. | n/a |
| *Ethical approval* | Name the institutional research board or ethics committee that approved the study and describe the participant-informed consent or the ethics committee waiver of informed consent. | 17 |
| **Open Science** | | |
| *Funding* | Give the source of funding and the role of the funders for the present study. | 17 |
| *Conflicts of interest* | Declare any conflicts of interest and financial disclosures for all authors. | 17 |
| *Protocol* | Indicate where the study protocol can be accessed or state that a protocol was not prepared. | 4, 5 |
| *Registration* | Provide registration information for the study, including register name and registration number, or state that the study was not registered. | 3 |
| *Data sharing* | Provide details of the availability of the study data. | 17 |
| *Code sharing* | Provide details of the availability of the analytical code. | 8, 17 |
| **Patient & Public Involvement** | | |
| *Patient & public involvement* | Provide details of any patient and public involvement during the design, conduct, reporting, interpretation, or dissemination of the study or state no involvement. | 17 |
| **Results** | | |
| *Participants* | Describe the flow of participants through the study, including the number of participants with and without the outcome and, if applicable, a summary of the follow-up time. A diagram may be helpful. | 10, Supplementary Figures S1 and S2 |
|  | Report the characteristics overall and, where applicable, for each data source or setting, including the key dates, key predictors (including demographics), treatments received, sample size, number of outcome events, follow-up time, and amount of missing data. A table may be helpful. Report any differences across key demographic groups. | 10, 11, Table 1 |
|  | For model evaluation, show a comparison with the development data of the distribution of important predictors (demographics, predictors, and outcome). | n/a |
| *Model development* | Specify the number of participants and outcome events in each analysis (e.g., for model development, hyperparameter tuning, model evaluation. | 7, 8 |
| *Model specification* | Provide details of the full prediction model (e.g., formula, code, object, application programming interface) to allow predictions in new individuals and to enable third-party evaluation and implementation, including any restrictions to access or re-use (e.g., freely available, proprietary). | Supplementary Figure S3 |
| *Model performance* | Report model performance estimates with confidence intervals, including for any key subgroups (e.g., sociodemographic). Consider plot to aid presentation. | 10, 11, Figures 2-3 |
|  | If examined, report results of any heterogeneity in model performance across clusters. See TRIPOD Cluster for additional details. | n/a |
| *Model updating* | Report the results from any model updating, including the updated model and subsequent performance. | n/a |
| **Discussion** | | |
| *Interpretation* | Give an overall interpretation of the main results, including issues of fairness in the context of the objectives and previous studies. | 12-14 |
| *Limitations* | Discuss any limitations of the study (such as a non-representative sample, sample size, overfitting, missing data) and their effects on any biases, statistical uncertainty, and generalizability. | 15, 16 |
| *Usability of the*  *model in the*  *context of current*  *care* | Describe how poor quality or unavailable input data (e.g., predictor values) should be assessed and handled when implementing the prediction model. | Supplementary Figure S3 |
|  | Specify whether users will be required to interact in the handling of the input data or use of the model, and what level of expertise is required of users. | Supplementary Figure S3 |
|  | Discuss any next steps for future research, with a specific view to applicability and generalizability of the model. | 14-16 |

## References

[1]	Leinert C, Brefka S, Fotteler ML, Mueller-Stierlin AS, Gebhard F, Rahbari N, et al. Standard-of-care vs expert-recommended discharge destinations for geriatric surgical inpatients: a prospective observational cohort study. Eur Geriatr Med 2025. https://doi.org/10.1007/s41999-025-01382-x.
